# Unilateral Versus Bilateral Cochlear Implants in Adults: A Cross-Sectional Questionnaire Study Across Multiple Hearing Domains

**DOI:** 10.3390/audiolres15010006

**Published:** 2025-01-20

**Authors:** Alessandra Pantaleo, Luigi Curatoli, Giada Cavallaro, Debora Auricchio, Alessandra Murri, Nicola Quaranta

**Affiliations:** 1 Otolaryngology Unit, Department of Traslational Medicine and Neuroscience-DiBrain, University of Bari, 70124 Bari, Italy; 2 Otolaryngology Unit, Madonna delle Grazie Hospital of Matera, 75100 Matera, Italy

**Keywords:** cochlear implants, speech perception, music perception, tinnitus, quality of life

## Abstract

Aim: The aim of this study was to assess the subjective experiences of adults with different cochlear implant (CI) configurations—unilateral cochlear implant (UCI), bilateral cochlear implant (BCI), and bimodal stimulation (BM)—focusing on their perception of speech in quiet and noisy environments, music, environmental sounds, people’s voices and tinnitus. Methods: A cross-sectional survey of 130 adults who had undergone UCI, BCI, or BM was conducted. Participants completed a six-item online questionnaire, assessing difficulty levels and psychological impact across auditory domains, with responses measured on a 10-point scale. Statistical analyses were performed to compare the subjective experiences of the three groups. Results: Patients reported that understanding speech in noise and tinnitus perception were their main concerns. BCI users experienced fewer difficulties with understanding speech in both quiet (*p* < 0.001) and noisy (*p* = 0.008) environments and with perceiving non-vocal sounds (*p* = 0.038) compared to UCI and BM users; no significant differences were found for music perception (*p* = 0.099), tinnitus perception (*p* = 0.397), or voice naturalness (*p* = 0.157). BCI users also reported less annoyance in quiet (*p* = 0.004) and noisy (*p* = 0.047) environments, and in the perception of voices (*p* = 0.009) and non-vocal sounds (*p* = 0.019). Tinnitus-related psychological impact showed no significant differences between groups (*p* = 0.090). Conclusions: Although speech perception in noise and tinnitus remain major problems for CI users, the results of our study suggest that bilateral cochlear implantation offers significant subjective advantages over unilateral implantation and bimodal stimulation in adults, particularly in difficult listening environments.

## 1. Introduction

Hearing disorders can significantly affect interpersonal communication, employment, education, and social participation, thereby influencing quality of life (QoL) [1]. Additionally, there is a strong association between sensorineural hearing loss (SNHL) and tinnitus [2].

As the prevalence of hearing loss increases [3], the demand for cost-effective interventions to mitigate its adverse effects increases. When hearing aids fail to provide sufficient understanding or acquisition of speech, cochlear implantation becomes necessary. Cochlear implants (CIs) are surgically implantable devices that bypass nonfunctional structures by directly stimulating the cochlear nerve [4], making them the treatment of choice for severe and profound bilateral hearing loss. Advancements in technology and knowledge about CI fitting, and the possibility to preserve residual hearing, have broadened the eligibility criteria for cochlear implantation in both adult and pediatric populations [5,6]. Consequently, various CI configurations are now feasible, including unilateral cochlear implantation (UCI), bilateral cochlear implantation (BCI), and bimodal stimulation (BM), which combines a cochlear implant in one ear with a hearing aid (HA) in the contralateral ear.

As a result, the decision-making process has become increasingly complex and requires in-depth discussions between patients, family members, and professionals. Although audiological measures such as speech recognition ability, assessed by word and sentence recognition scores (in silence or in noise), remain the most widely used [7], there is a growing need for tools that aim to know the patients’ perceptions on their condition and treatments. In this regard, patient-reported outcome measures (PROMs), are essential tools to assess the multidimensional effects of CIs [8]. These instruments evaluate domains such as speech understanding in different settings and overall quality of life (QoL), providing insights that complement objective audiometric assessments. Importantly, PROMs facilitate the identification of physical challenges, including communication difficulties and emotional wellbeing, which may not be apparent from clinical testing. Several self-report questionnaires, such as the HISQUI [9] and the SSQ [10], have been widely used in the clinical settings. More recently, Gomersall et al. [11] introduced a six-item questionnaire focusing on areas of potential difficulty for CI users, as well as the tinnitus-specific question. The questionnaire arose from the need to overcome the existing limitations of traditional self-report measures for assessing tinnitus in people with a CI compared to the general population. Indeed, CI users typically experience more severe and prolonged hearing loss than those in validation studies and reports of tinnitus can vary significantly when the symptom is assessed in isolation from questions about other hearing problems [11].

The purpose of our study was to assess, using the six-item questionnaire developed by Gomersall et al. [11], the impact of deafness and the self-reported benefits across different cochlear implant user profiles (UCI, BCI, and BM) in various domains commonly reported as challenging for CI users.

## 2. Materials and Methods

### 2.1. Ethical Considerations

The study was conducted in accordance with the ethical principles of the Declaration of Helsinki. Ethical approval was obtained from the local ethics committee (Prot. N 006712/02/08/2021). Informed consent was obtained from all participants via a dedicated section in the Google form used for data collection. Participants were informed of the purpose of the study, their right to withdraw at any time, and that their data would be anonymized in accordance with applicable privacy regulations. By completing the consent section of the Google Form, participants consented to the collection and use of their data for research purposes.

### 2.2. Study Design

This study used a cross-sectional survey design using the Google Forms platform. The survey targeted people with hearing loss who were members of the online support group “Affrontiamo la sordità insieme—Let’s face deafness together”. Eligible participants were aged 18 years or older and either English- or Italian-speaking, with no disability other than hearing loss (e.g., visual impairment, cognitive impairment).

### 2.3. Survey Administration

The survey consisted of two parts:

▪ Demographic and General Information: Participants were first asked to provide details regarding their gender, age, country and spoken language, presence of tinnitus, and use of hearing aids (HA) or cochlear implants (CIs), including specific types of rehabilitation (UCI, BCI, BM).
▪ Six-item Questionnaire: Participants completed the questionnaire developed by Gomersall et al. [11] (Appendix A). The questionnaire evaluates hearing performance and the perceived impact on one’s life in six areas (henceforth referred to as domains) which are reported as problematic in CI users:
  ○ Speech perception in a quiet room;
  ○ Speech perception in noisy environment;
  ○ Perception of music;
  ○ Perception of tinnitus;
  ○ Naturalness of voices;
  ○ Naturalness of environmental sounds.

For each domain, “Part A” of the questionnaire requests the subject to quantify the level of difficulty for that domain, while “Part B” of the questionnaire requests the subject to rate the perceived psychological impact of that difficulty. All responses were based on the hearing status of the participants with an active CI and were given on a Visual Analogue Scale (VAS) ranging from 0 to 10 points, where 0 represents the lowest level of difficulty/ psychological impact, and 10 represents the highest.

### 2.4. Statistical Analysis

Data were collected and divided into different sets. Gender and mean age were reported. The mean score for each question and its relative standard deviation were also recorded. The answers from Group A (UCI users), B (BCI users) and C (BM users) had non-normal distributions (Shapiro–Wilk, *p* < 0.001), so the comparison per item was conducted using the Kruskal–Wallis test (with the Šidák correction method and Dunn’s multiple comparison method, adjusted for multiple comparison); the significance level *p* was set lower than 0.05 in all cases.

## 3. Results

A total of 214 questionnaires were returned within 8 weeks. Only complete questionnaires were considered and only responses from participants with CI (UCI, BCI, BM) were included in the study group. Table 1 provides data on those who responded to the questionnaire. A total of 130 participants were included in the study group: 94 were female (72.3%) and 36 were male (27.7%). The mean age was 50.1 years (SD, 10.5; range, 15–80 years). Seventy-six participants (58.5%) were Italian native speakers, while fifty-four participants (41.5%) were English native speakers. Forty-one participants had a unilateral cochlear implant (UCI, Group A), forty-six participants had a bilateral cochlear implant (BCI, Group B), and forty-three had bimodal stimulation (BM, Group C). No significant differences in terms of age and gender were found among the three groups (*p* = 0.373 and *p* = 0.400, respectively; one-way ANOVA and chi-square test of independence).

**Figure 1:**
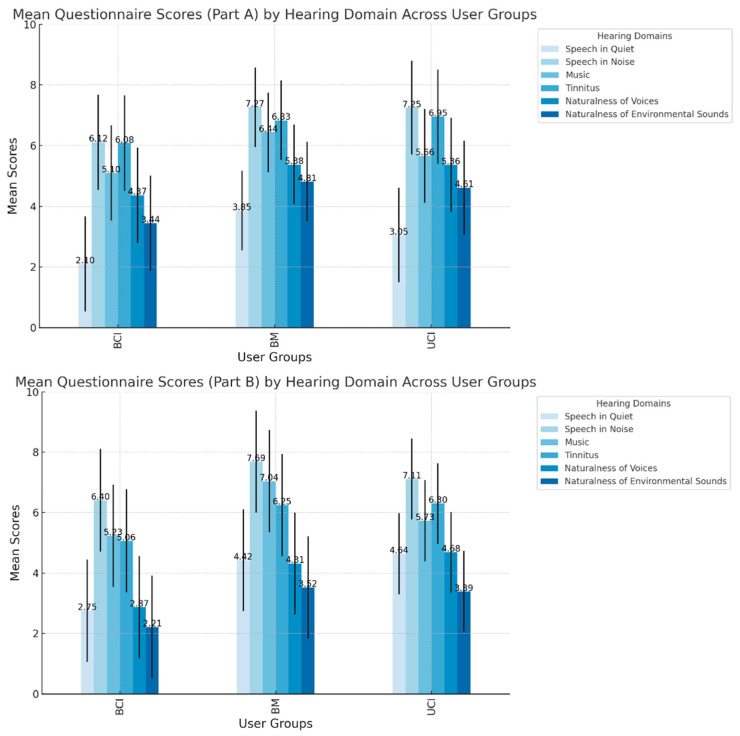
Mean questionnaire scores by hearing domain across user group. Bar chart showing mean scores of the questionnaire for UCI, BCI, and BM users. Part A assesses auditory difficulty, while Part B evaluates psychological impact. Error bars represent the standard deviation.

The mean scores and standard deviations (SD) for all the questions are shown in Table 2, and a visual summary is provided in Figure 1. Among all groups (UCI, BCI, BM), the most significant difficulty was understanding other people speaking with background noise, with VAS scores of 7.25, 6.12, and 7.27 for UCI, BCI, and BM, respectively, in Part A, and 7.11, 6.40, and 7.69 in Part B (*p* always < 0.001; Kruskall–Wallis, adjusted for multiple comparison). The second most impacted domain was tinnitus perception, with mean VAS scores of 6.93, 6.08, and 6.83 for UCI, BCI, and BM, respectively, in Part A, and 6.30, 5.06, and 6.25 in Part B.

**Figure 2:**
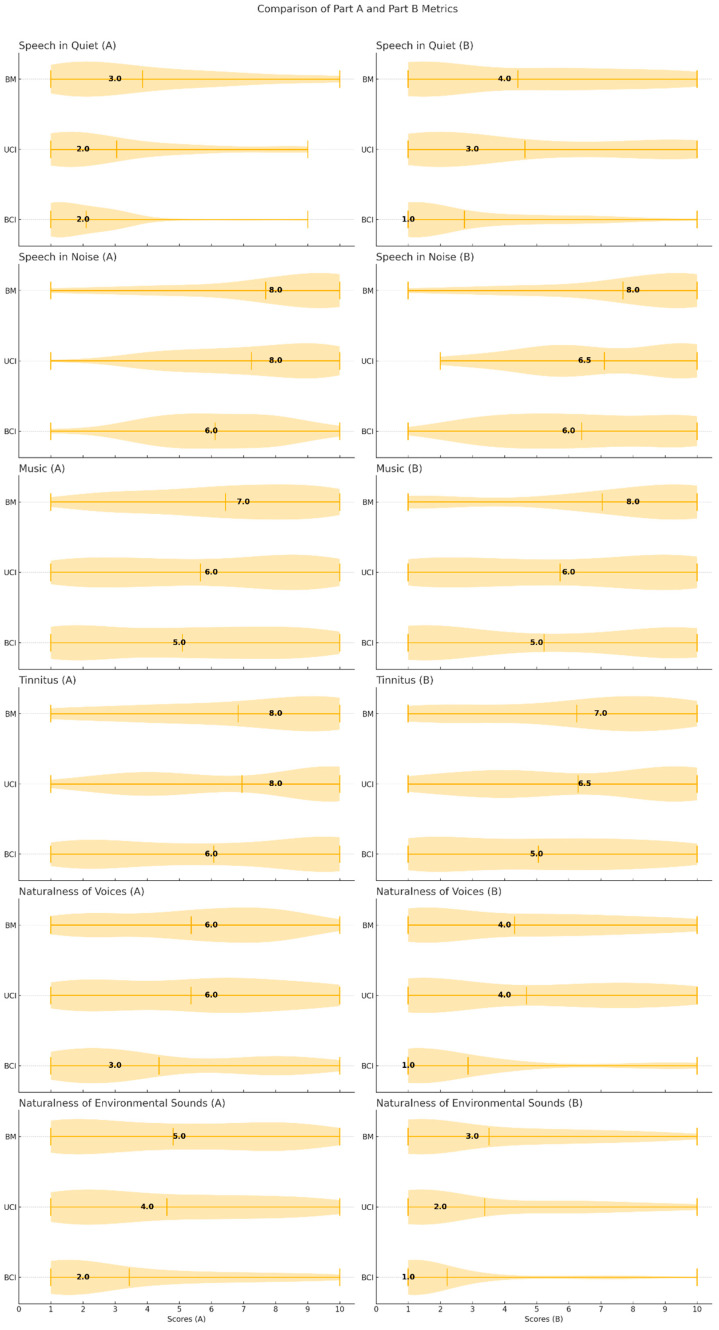
Violin plots comparing scores for each item across UCI (unilateral cochlear implant), BM (bimodal stimulation), and BCI (bilateral cochlear implant) users, divided into Part A (**left**) and Part B (**right**). Medians are highlighted in black.

When analyzing responses to individual questions across the three groups, focusing on Part A of the questionnaire (difficulty levels), BCI users reported fewer difficulties than UCI and BM users in understanding speech in a quiet room (*p* < 0.001, h = 13.18 in Kruskal–Wallis, with a similar median value for BCI and UCI) and background noise settings (*p* = 0.008, h = 9.95). Additionally, BCI users demonstrated a more natural perception of non-vocal sounds, such as running water (*p* = 0.038, h = 6.16); no significant differences were found in music perception (*p* = 0.099, h = 4.65), tinnitus perception (*p* = 0.397, h = 1.82), and naturalness of people’s voices (*p* = 0.157, h = 3.51). Moreover, in the assessment of responses related to Part B of the questionnaire (psychological impact), we found that BCI users were significantly less impaired (i.e., better hearing-QoL) in most domains, including speech perception in quiet (*p* = 0.004, h = 10.62) and noisy environments (*p* = 0.047, h = 6.33), as well as naturalness of people’s voices (*p* = 0.009 h = 9.34) and non-vocal sounds (*p* = 0.019, h = 7.50). No significant difference was found in tinnitus-related psychological impact between the groups (*p* = 0.090, h = 4.83). Figure 2 illustrates the distribution of responses for each item across the groups, with medians highlighted.

## 4. Discussion

Despite advancements in cochlear implant technology, some hearing domains remain challenging for CI users, regardless of the auditory rehabilitation profile. It is therefore becoming increasingly important to identify tools that can more clearly indicate the difficulties that CI users face in their daily lives, which can then be used to guide the rehabilitation process in a patient-centered way. For this reason, self-report measures are increasingly being used alongside standard objective audiological measures. Among these, the six-item questionnaire developed by Gomersall et al. [11], originally designed to better define the impact of tinnitus on CI users, has the advantage of combining the assessment of auditory challenges across multiple domains—including music perception, environmental sounds, and voice naturalness—with a direct assessment of the psychological impact of these difficulties on daily life, making it particularly useful for personalized interventions. Moreover, its brevity and simplicity enhance its practicality in clinical and research settings, as it is less time-consuming than the HISQUI or SSQ, and the inclusion of a visual analog scale (VAS) further improves its accessibility, allowing participants to intuitively convey their experiences and concerns.

In our study, we found the six-item questionnaire to be very useful in assessing the subjective benefits in both Italian- and English-speaking patients with different hearing rehabilitation modalities (UCI, BCI, and BM). A common difficulty across all user groups (UCI, BCI, and BM) was understanding speech in noisy environments, although BCI users reported fewer difficulties in both quiet and noisy settings compared to UCI and BM users. Indeed, several studies have highlighted the advantages of binaural stimulation, including both bilateral cochlear implants and bimodal stimulation, over unilateral implantation. Bimodal hearing offers advantages over unilateral implantation for speech recognition in both quiet and noisy environments [12,13,14,15,16,17,18]. Similarly, bilateral cochlear implant users experience significant benefits in speech recognition in these settings compared to those with a unilateral implant [13,18,19,20]. Overall, research suggests comparable benefits for bilateral CI and bimodal users in these listening conditions [13,14,21,22]. Potts and Litovsky [23] examined the outcomes of patients who switched from a bimodal to a bilateral CI configuration; out of four patients, two showed better speech recognition and all showed better sound localization and higher performance, as assessed by the SSQ questionnaire, compared to bimodal stimulation. In another within-subject comparison of bimodal and bilateral CI outcomes by Luntz et al. [21], patients showed better speech processing and localization in complex listening environments and higher overall SSQ scores with the bilateral CI. Similarly, Yawn et al. [24] found that in patients with bimodal hearing configuration and substantial residual hearing in the non-CI ear, bilateral cochlear implantation improved audiological performance and subjective quality of life, as reflected by higher SSQ scores.

Another common problem across all groups in our study was tinnitus perception. Indeed, tinnitus continues to be a problem for a significant proportion of cochlear implant users, as revealed by a recent study by Gomersall et al. [11] and previous studies, although severity is not often detailed [25,26]. Research has shown that the use of CI increases the activity of the peripheral auditory system, providing better access to external sounds that either mask or divert the patient’s attention from the perception of tinnitus [27]. Summerfield et al. [28] and van Zon et al. [29] found that the perception of tinnitus and onset of newly induced tinnitus did not differ significantly between simultaneous bilateral CI, sequential CI, and UCI. More recently, Quaranta et al. [2] evaluated the effect of CI on tinnitus in patients with UCI, BCI, and BM, and found no statistically significant differences in THI score, between unilateral and bilateral electrical stimulation and in its impact on quality of life among BCI, UCI, and BM users. Consistent with these findings, our within-group analysis identified tinnitus as the second most affected area in each group, but statistical analysis revealed no significant differences in tinnitus perception and its impact on QoL between UCI, BCI, and BM users.

Moreover, cochlear implant users generally report poor music quality due to the device’s inherent limitations in transmitting signals related to fine temporal structures, which are essential for accurate perception of musical pitch and timbre [30,31]. In our study, no statistically significant differences were found between UCI, BCI, and BM users. However, when assessing responses related to psychological impact Part B), we found that BCI users were less impaired overall (i.e., better QoL) in most domains, including following music.

Overall, bilateral cochlear implants and bimodal stimulation have shown improvements over unilateral cochlear implants [32,33,34]. However, it remains difficult to determine whether people with residual hearing in the contralateral ear after cochlear implantation receive greater benefit from bimodal hearing devices or bilateral implants. Bilateral implants may provide greater benefit because the binaural system is better able to compare inputs when they coincide, compared with different inputs. On the other hand, combining a hearing aid with a cochlear implant may offer potential benefits in music perception and sound quality due to the complementarity of low-frequency residual hearing and high-frequency electrical hearing [32].

With regard to environmental sounds (i.e., non-speech, non-musical auditory cues that provide information about the surrounding environment), better perception was found for BCI users than for BM and UCI users. Environmental sounds play an important role in the independence, safety, quality of life, and well-being of CI users [35,36,37] by aiding spatial orientation, signaling potential hazards, and promoting a sense of connectedness to the external environment. Indeed, as shown in other research articles, environmental sound awareness is one of the main benefits of cochlear implantation [36,38]. However, the large individual variability and methodological limitations that exist in the assessment of environmental sound perception may potentially obscure the potential benefits of CI in this regard. Indeed, a recent systematic review by Shafiro et al. [39] revealed generally poor levels of environmental sound identification and no apparent improvement in group performance after implantation compared to the baseline. Nevertheless, there was a trend of bimodal and bilateral CI users outperforming unilateral CI users in terms of environmental sound perception [40,41] and our findings are consistent with those of previous studies.

We acknowledge several limitations of the present study. The six-item questionnaire was originally designed for a different purpose than the one used in our study (i.e., to define the perception and impact of tinnitus in CI users in relation to specific auditory domains) and has not yet been validated in the Italian language. Although the use of an online survey facilitated the participation of a large and geographically diverse sample, including respondents from Europe, North America, and Oceania, data from some areas such as Africa and Asia were missing. Furthermore, reliance on self-reported data from patients rather than CI professionals limited the availability of critical information, including objective audiological measurements, specific types of devices used, and the duration of deafness prior to implantation. The absence of these objective data precluded correlation analyses between subjective reports and measurable audiological outcomes, introducing a potential bias into our results. In particular, the lack of information on contralateral residual hearing makes it difficult to definitively assess the benefits of bilateral cochlear implantation over bimodal stimulation.

However, we believe that this survey provides a snapshot of the current problems faced by cochlear implant recipients worldwide and, more generally, of the advantages of binaural stimulation, including both bilateral implantation and bimodal stimulation, over unilateral implantation.

## 5. Conclusions

In conclusion, despite its limitations, this study provides valuable insights into the subjective benefits experienced by patients with different cochlear implant configurations in different auditory domains, including both English and Italian speakers. Although speech perception in noise and tinnitus remain major problems for CI users, the results of our study suggest that bilateral cochlear implantation offers significant subjective advantages over unilateral implantation and bimodal stimulation in adults, particularly in difficult listening environments.

## Figures and Tables

**Table 1:** Study group data. The table summarizes the mean age, gender distribution, and the number of participants using a unilateral cochlear implant (UCI), a bilateral cochlear implant (BCI), or bimodal stimulation (BM).

	Italian Responders (n = 76)	English Responders (n = 54)	Total (n = 130)
Mean Age	46.8	55.0	50.1
Male	23	13	36
Female	53	41	94
Unilateral Cochlear Implant (UCI, Group A)	27	14	41
Bilateral Cochlear Implant (BCI, Group B)	24	22	46
Bimodal Stimulation (BM, Group C)	25	18	43

**Table 2:** Mean scores and standard deviations across domains. Mean scores and standard deviations (SD) from the six-item questionnaire are presented for unilateral cochlear implant (UCI), bilateral cochlear implant (BCI), and bimodal stimulation (BM) users. The questions assess levels of difficulty (Part A) and perceived psychological impact (Part B) across different hearing domains.

Question		Group A UCI (Mean; SD)	Group B BCI (Mean; SD)	Group C BM (Mean; SD)
Speech in Quiet Setting	A	3.05 (2.49)	2.10 (1.55)	3.85 (2.70)
	B	4.64 (3.45)	2.75 (2.440)	4.42 (3.29)
Speech in Noisy Setting	A	7.25 (2.33)	6.12 (2.15)	7.27 (1.91)
	B	7.11 (2.51)	6.40 (2.68)	7.69 (2.66)
Music	A	5.66 (3.16)	5.10 (3.18)	6.44 (2.70)
	B	5.73 (3.42)	5.23 (3.77)	7.04 (3.35)
Tinnitus	A	6.96 (3.10)	6.08 (3.48)	6.83 (3.02)
	B	6.30 (3.23)	5.06 (3.18)	6.25 (3.06)
Naturalness of voices	A	5.36 (3.05)	4.37 (3.01)	5.38 (2.68)
	B	4.68 (3.43)	2.87 (2.87)	4.31 (3.16)
Naturalness of Environmental Sound	A	4.61 (3.01)	3.44 (2.81)	4.81 (3.14)
	B	3.39 (2.90)	2.21 (2.21)	3.52 (2.86)

## Data Availability

The raw data supporting the conclusions of this article will be made available by the authors on request.

## Appendix A

**Table A1:** The six-item questionnaire proposed by Gomersall et al. [11].

Question	Caption	Scoring (VAS)
IA	How difficult is it for you to understand a single person speaking in a quiet room?	0–10
IB	How much does this upset/annoy/worry you?	0–10
IIA	How difficult is it for you to understand a single person speaking in background noise?	0–10
IIB	How much does this upset/annoy/worry you?	0–10
IIIA	How difficult is it for you to follow music?	0–10
IIIB	How much does this upset/annoy/worry you?	0–10
IVA	How often are you aware of any buzzing, ringing or other noises in your head or ears (tinnitus)?	0–10
IVB	How much does this upset/annoy/worry you?	0–10
VA	How natural are people’s voices?	0–10
VB	How much does this upset/annoy/worry you?	0–10
VIA	How natural are everyday sounds other than people talking (for example running water)?	0–10
VIB	How much does this upset/annoy/worry you?	0–10

Note: VAS (Visual Analogue Scale): 0 is the lowest level of difficulty/psychological impact, and 10 represents the highest.

## References

[1] Brodie A., Smith B., Ray J. (2018). The Impact of Rehabilitation on Quality of Life after Hearing Loss: A Systematic Review. Eur. Arch. Otorhinolaryngol..

[2] Quaranta N., Baguley D., Fanizzi P., Murri A., Pontillo V., Cutler J.M., Cavallaro G. (2023). The Effect of Cochlear Implant and Bimodal Stimulation on Tinnitus: A Multinational Survey. Acta Otolaryngol..

[3] Goman A.M., Reed N.S., Lin F.R. (2017). Addressing Estimated Hearing Loss in Adults in 2060. JAMA Otolaryngol. Head Neck Surg..

[4] Naples J.G., Ruckenstein M.J. (2020). Cochlear Implant. Otolaryngol. Clin. N. Am..

[5] Dillon M.T., Kocharyan A., Daher G.S., Carlson M.L., Shapiro W.H., Snapp H.A., Firszt J.B. (2022). American Cochlear Implant Alliance Task Force Guidelines for Clinical Assessment and Management of Adult Cochlear Implantation for Single-Sided Deafness. Ear Hear..

[6] Park L.R., Griffin A.M., Sladen D.P., Neumann S., Young N.M. (2022). American Cochlear Implant Alliance Task Force Guidelines for Clinical Assessment and Management of Cochlear Implantation in Children with Single-Sided Deafness. Ear Hear..

[7] Adunka O.F., Gantz B.J., Dunn C., Gurgel R.K., Buchman C.A. (2018). Minimum Reporting Standards for Adult Cochlear Implantation. Otolaryngol. Head. Neck Surg..

[8] McRackan T.R., Bauschard M., Hatch J.L., Franko-Tobin E., Droghini H.R., Velozo C.A., Nguyen S.A., Dubno J.R. (2018). Meta-Analysis of Cochlear Implantation Outcomes Evaluated With General Health-Related Patient-Reported Outcome Measures. Otol. Neurotol..

[9] Amann E., Anderson I. (2014). Development and Validation of a Questionnaire for Hearing Implant Users to Self-Assess Their Auditory Abilities in Everyday Communication Situations: The Hearing Implant Sound Quality Index (HISQUI19). Acta Otolaryngol..

[10] Gatehouse S., Noble W. (2004). The Speech, Spatial and Qualities of Hearing Scale (SSQ). Int. J. Audiol..

[11] Gomersall P.A., Baguley D.M., Carlyon R.P. (2019). A Cross-Sectional Questionnaire Study of Tinnitus Awareness and Impact in a Population of Adult Cochlear Implant Users. Ear Hear..

[12] Crew J.D., Galvin J.J., Fu Q.-J. (2016). Perception of Sung Speech in Bimodal Cochlear Implant Users. Trends Hear..

[13] Gifford R.H., Dorman M.F., Sheffield S.W., Teece K., Olund A.P. (2014). Availability of Binaural Cues for Bilateral Implant Recipients and Bimodal Listeners with and without Preserved Hearing in the Implanted Ear. Audiol. Neurootol..

[14] Gifford R.H., Dorman M.F. (2019). Bimodal Hearing or Bilateral Cochlear Implants? Ask the Patient. Ear Hear..

[15] Kessler D.M., Ananthakrishnan S., Smith S.B., D’Onofrio K., Gifford R.H. (2020). Frequency Following Response and Speech Recognition Benefit for Combining a Cochlear Implant and Contralateral Hearing Aid. Trends Hear..

[16] Kessler D.M., Wolfe J., Blanchard M., Gifford R.H. (2020). Clinical Application of Spectral Modulation Detection: Speech Recognition Benefit for Combining a Cochlear Implant and Contralateral Hearing Aid. J. Speech Lang. Hear. Res..

[17] Neuman A.C., Waltzman S.B., Shapiro W.H., Neukam J.D., Zeman A.M., Svirsky M.A. (2017). Self-Reported Usage, Functional Benefit, and Audiologic Characteristics of Cochlear Implant Patients Who Use a Contralateral Hearing Aid. Trends Hear..

[18] van Hoesel R.J.M. (2012). Contrasting Benefits from Contralateral Implants and Hearing Aids in Cochlear Implant Users. Hear. Res..

[19] Buss E., Dillon M.T., Rooth M.A., King E.R., Deres E.J., Buchman C.A., Pillsbury H.C., Brown K.D. (2018). Effects of Cochlear Implantation on Binaural Hearing in Adults With Unilateral Hearing Loss. Trends Hear..

[20] Litovsky R., Parkinson A., Arcaroli J., Sammeth C. (2006). Simultaneous Bilateral Cochlear Implantation in Adults: A Multicenter Clinical Study. Ear Hear..

[21] Luntz M., Egra-Dagan D., Attias J., Yehudai N., Most T., Shpak T. (2014). From Hearing with a Cochlear Implant and a Contralateral Hearing Aid (CI/HA) to Hearing with Two Cochlear Implants (CI/CI): A within-Subject Design Comparison. Otol. Neurotol..

[22] Schafer E.C., Amlani A.M., Seibold A., Shattuck P.L. (2007). A Meta-Analytic Comparison of Binaural Benefits between Bilateral Cochlear Implants and Bimodal Stimulation. J. Am. Acad. Audiol..

[23] Potts L.G., Litovsky R.Y. (2014). Transitioning from Bimodal to Bilateral Cochlear Implant Listening: Speech Recognition and Localization in Four Individuals. Am. J. Audiol..

[24] Yawn R.J., O’Connell B.P., Dwyer R.T., Sunderhaus L.W., Reynolds S., Haynes D.S., Gifford R.H. (2018). Bilateral Cochlear Implantation Versus Bimodal Hearing in Patients With Functional Residual Hearing: A Within-Subjects Comparison of Audiologic Performance and Quality of Life. Otol. Neurotol..

[25] Ruckenstein M.J., Hedgepeth C., Rafter K.O., Montes M.L., Bigelow D.C. (2001). Tinnitus Suppression in Patients with Cochlear Implants. Otol. Neurotol..

[26] Souliere C.R.J., Kileny P.R., Zwolan T.A., Kemink J.L. (1992). Tinnitus Suppression Following Cochlear Implantation. A Multifactorial Investigation. Arch. Otolaryngol. Head. Neck Surg..

[27] Quaranta N., Wagstaff S., Baguley D.M. (2004). Tinnitus and Cochlear Implantation. Int. J. Audiol..

[28] Quentin Summerfield A., Barton G.R., Toner J., McAnallen C., Proops D., Harries C., Cooper H., Court I., Gray R., Osborne J. (2006). Self-Reported Benefits from Successive Bilateral Cochlear Implantation in Post-Lingually Deafened Adults: Randomised Controlled Trial. Int. J. Audiol..

[29] van Zon A., Smulders Y.E., Ramakers G.G.J., Stegeman I., Smit A.L., Van Zanten G.A., Stokroos R.J., Hendrice N., Free R.H., Maat B. (2016). Effect of Unilateral and Simultaneous Bilateral Cochlear Implantation on Tinnitus: A Prospective Study. Laryngoscope.

[30] McDermott H.J. (2004). Music Perception with Cochlear Implants: A Review. Trends Amplif..

[31] Riley P.E., Ruhl D.S., Camacho M., Tolisano A.M. (2018). Music Appreciation after Cochlear Implantation in Adult Patients: A Systematic Review. Otolaryngol. Head. Neck Surg..

[32] Ching T.Y.C., van Wanrooy E., Dillon H. (2007). Binaural-Bimodal Fitting or Bilateral Implantation for Managing Severe to Profound Deafness: A Review. Trends Amplif..

[33] D’Onofrio K.L., Gifford R.H. (2021). Bimodal Benefit for Music Perception: Effect of Acoustic Bandwidth. J. Speech Lang. Hear. Res..

[34] El Fata F., James C.J., Laborde M.-L., Fraysse B. (2009). How Much Residual Hearing Is “useful” for Music Perception with Cochlear Implants?. Audiol. Neurootol..

[35] Hamel B.L., Vasil K., Shafiro V., Moberly A.C., Harris M.S. (2020). Safety-Relevant Environmental Sound Identification in Cochlear Implant Candidates and Users. Laryngoscope.

[36] McRackan T.R., Hand B.N., Velozo C.A., Dubno J.R. (2019). Development of the Cochlear Implant Quality of Life Item Bank. Ear Hear..

[37] Vasil K.J., Lewis J., Tamati T., Ray C., Moberly A.C. (2020). How Does Quality of Life Relate to Auditory Abilities? A Subitem Analysis of the Nijmegen Cochlear Implant Questionnaire. J. Am. Acad. Audiol..

[38] McRackan T.R., Velozo C.A., Holcomb M.A., Camposeo E.L., Hatch J.L., Meyer T.A., Lambert P.R., Melvin C.L., Dubno J.R. (2017). Use of Adult Patient Focus Groups to Develop the Initial Item Bank for a Cochlear Implant Quality-of-Life Instrument. JAMA Otolaryngol. Head. Neck Surg..

[39] Shafiro V., Luzum N., Moberly A.C., Harris M.S. (2021). Perception of Environmental Sounds in Cochlear Implant Users: A Systematic Review. Front. Neurosci..

[40] McMahon K.R., Moberly A.C., Shafiro V., Harris M.S. (2018). Environmental Sound Awareness in Experienced Cochlear Implant Users and Cochlear Implant Candidates. Otol. Neurotol..

[41] Nyirjesy S., Rodman C., Tamati T.N., Moberly A.C. (2020). Are There Real-World Benefits to Bimodal Listening?. Otol. Neurotol..

